# Artificial Neural Network (ANN) Modeling for Predicting Performance of SBS Modified Asphalt

**DOI:** 10.3390/ma15238695

**Published:** 2022-12-06

**Authors:** Ke Zhong, Qiao Meng, Mingzhi Sun, Guobao Luo

**Affiliations:** 1 Research Institute of Highway Ministry of Transport, Beijing 100088, China; 2 Key Laboratory of Transport Industry of Road Structure and Material, Beijing 100088, China; 3 School of Civil Engineering, Chongqing Jiaotong University, Chongqing 400074, China

**Keywords:** SBS modified asphalt, ATR-FTIR, artificial neural networks, SBS content, performance parameters

## Abstract

Due to the superiorities of Styrene butadiene styrene (SBS) modified asphalt, it is widely used in civil engineering application. Meanwhile, accurately predicting and obtaining performance parameters of SBS modified asphalt in unison is difficult. At present, it is essential to discover an accurate and simple method between the input and output data. ANNs are used to model the performance and behavior of materials in place of conventional physical tests because of their adaptability and learning. The objective of this study discussed the application of ANNs in determining performance of SBS modified asphalt, based on attenuated total reflection Fourier transform infrared spectroscopy (ATR-FTIR) tests. A total of 150 asphalt mixtures were prepared from three matrix asphalt, two SBS modifiers and five modifier dosages. With the most suitable algorithm and number of neurons, an ANN model with seven hidden neurons was used to predict SBS content, needle penetration and softening point by using infrared spectral data of different modified asphalts as input. The results indicated that ANN-based models are valid for predicting the performance of SBS modified asphalt. The coefficient of determination (R^2^) of SBS content, softening point and penetration prediction models with the same grade of asphalt exceeded 99%, 98% and 96%, respectively. It can be concluded that ANNs can provide well-satisfied regression models between the SBS content and infrared spectrum statistics sets, and the precision of penetration and softening point model founded by the same grade of asphalt is high enough to can meet the forecast demand.

## 1. Introduction

With the dramatic increase in modern road traffic flow, frequency and axle weight of vehicles, more rigorous requirements have been placed on the performance and durability of pavement materials. Over decades of research, it has been found that polymer modifiers can effectively improve the performance of asphalt and enhance the durability of road materials [1,2,3,4]. Of which, SBS modifiers in particular are the best [5,6,7]. Ease of use and stability of performance are the advantages of using modified asphalt on road construction sites. However, the problem with modified asphalt is that the users are unaware of the base asphalt, the types and dosages of the modifiers; moreover, the product has a risk of segregation. Therefore, it is necessary to obtain accurate performance parameters of SBS modified asphalt, which can help researchers to carry out the mixture design or inspectors to test the quality of asphalt. Nevertheless, there are many performance parameters of modified asphalt, which would require a lot of time and effort if tested by indoor tests, and the test results also depend on the skill of the tester.

The combination of machine learning algorithms and vibrational spectroscopy has been a widely used analytical technique in the last decade [8,9,10,11]. Li et al. developed a calibration model for total oil acid number by partial least squares (PLS) combined with mid-infrared spectroscopy [12]. The predictive power of IR techniques has also been explored. Yuan et al. developed a strategy for classifying and identifying various residual oils using partial least squares regression and ATR-FTIR techniques to develop calibration models for physical and chemical indicators of three residual oils [13]. Kovalenko et al. developed a calibration model for estimating amino acid composition in soybean by PLS and support vector machine (SVM) regression methods of near infrared (NIR) spectroscopy [14]. The correlation between leaf water content and diffuse reflectance spectroscopy was investigated by Jin et al. Three multivariate calibration models including PLS, least squares svm (LSSVR) and radial basis function (RBF) neural network were developed to determine the leaf water content [15]. Wang Han et al. established a model for calculating the penetration, softening point and ductility with SBS content and furfural extract oil content, and the error between the calculated and actual values of this model can be controlled at about 5% [16].

The quality of modified asphalt can be improved by rapid measurement of SBS content, penetration and softening point. Since SBS has a distinct infrared characteristic peak in modified asphalt, this provides a good technical basis for the determination of SBS content. Currently, the standard curve method based on infrared spectroscopy is the main method for rapid determination of SBS content [17,18]. Sun et al. pointed out that the characteristic peaks of SBS at 699 cm^−1^ and 966 cm^−1^ and the characteristic peak of matrix asphalt at 810 cm^−1^ can be used as a qualitative and quantitative basis for SBS content determination [19]. The standard curve method has the advantages of high measurement accuracy and simple operation. However, the standard curves should be different for different types of SBS modified asphalt. Therefore, the standard curves must be prepared in advance according to the material of the specific project. In addition to this, soft computing also has an impact on neural network algorithms. M. Civera et al. investigated the feasibility of a bispectral damage localization method and showed that the best performing neural network consisted of a hidden layer of 26 neurons, which achieved an overall accuracy of about 80% in a training set of 1500 cases and could effectively predict damage problems of structures [20]. By comparing empirical hair, the regression method and software computation under as-built estimation (EAC) techniques, we found that there are limitations in prediction based on AI techniques, and the use of deep learning algorithms can effectively solve such problems [21].

Currently, the performance parameters of asphalt are tested according to industry standards, and it takes a lot of time and effort to prepare and fabricate the samples. The analysis shows that the basic properties of asphalt, such as penetration, softening point and other performance parameters are related to the type and content of functional groups, which can be quickly determined by advanced machine learning algorithms, such as infrared spectroscopy, to establish a predictive model of infrared spectral profiles and asphalt properties. While the existing research results mainly focus on the field of matrix asphalt [22], with the widespread use of SBS modified asphalt, an artificial neural network model can also be a rapid determination method for the performance of SBS modified asphalt.

Thus, this paper constructs an artificial neural network prediction model of modified asphalt infrared spectral data with SBS modifier admixture, needle penetration and softening point, and optimizes the accuracy of the model by comparing the differences between the predicted and actual values. It is expected that the performance of SBS modified asphalt can be quickly determined and the quality of the material can be effectively monitored in practical projects.

## 2. Experimental Procedures

### 2.1. Materials

**Figure 1:**
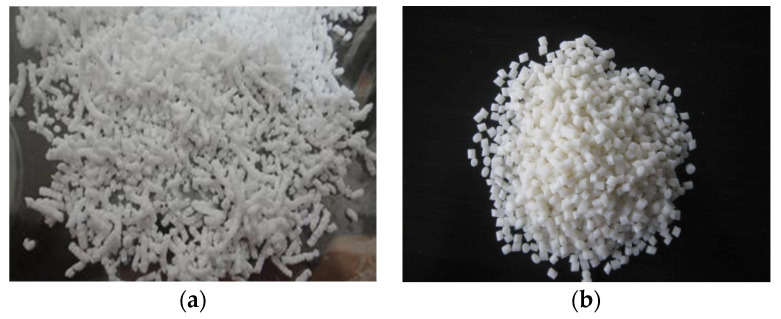
SBS modifiers. (**a**) YH791; (**b**) BM4302.

Three kinds of heavy traffic road petroleum asphalt, referred to Sinopec, Cnooc and Shell in this paper, were used raw materials for the test, the related performance is shown in Table 1. SBS modifiers are domestic road-type asphalt modifiers, one of which is linear YH791 and the other is star BM4302, and the appearance of the modifiers is shown in Figure 1. The SBS properties are shown in Table 2. The dosage of modifiers is 0, 2%, 3%, 4%, 5% and 6%, respectively.

### 2.2. Sample Preparation and Test Method

The SBS modified bitumen was prepared using a high-speed shear by heating three types of matrix bitumen to 170 °C and adding linear and star-shaped SBS modifiers at 0%, 2%, 3%, 4%, 5% and 6% modifiers, respectively. The shear rate of the high-speed shear was set at 6000 rpm, the temperature was 170 °C, and the shear time was 60 min. Thirty-six sets of samples were prepared according to JTG E20-2011 [23] specifications.

The penetration and softening point of modified asphalt were tested in 36 sets of asphalt samples with reference to JTG E20-2011. ATR-FTIR was used to acquire the infrared spectra of samples, and the range of infrared spectra was set from 4000 to 400 cm^−1^, which was accumulated by 16 scanning resolutions of 0.5 cm^−1^ with a total scanning time of 20 s.

**Figure 2:**
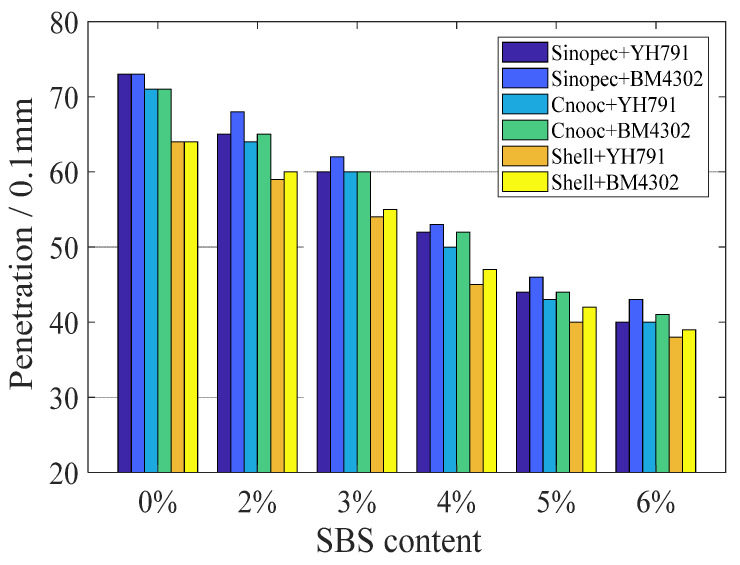
Penetration at 25 °C of all samples.

**Figure 3:**
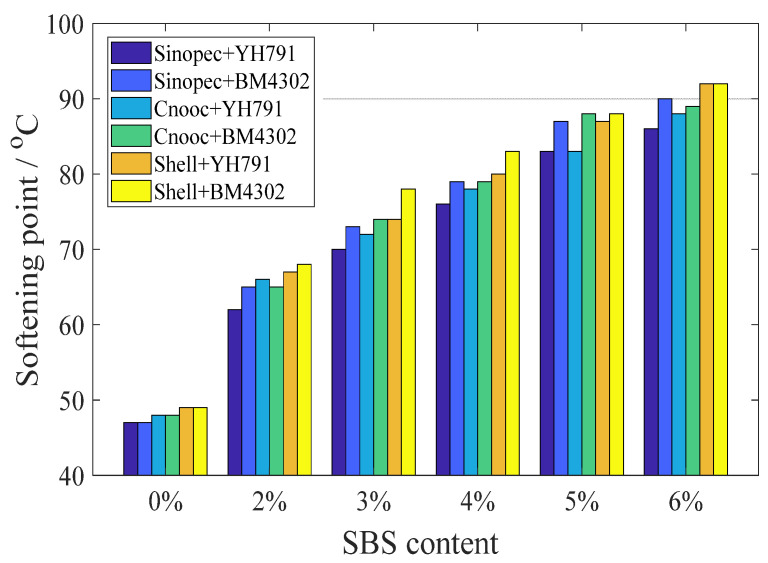
Softening point of all samples.

From Figure 2 and Figure 3, it can be seen that with the increase in SBS dosage, the penetration of the specimens decreased significantly, while the softening point showed the opposite trend. The results show that the viscosity and softening point of the asphalt increase when the modifier absorbs the lighter substances in the asphalt, while the penetration is the reverse. However, the ratio of the four components is different for different types of matrix asphalt. When the dosage of modifiers is the same, the less the component, the greater the softening point of the modified matrix asphalt, and the smaller the penetration as well. Therefore, the type of matrix asphalt and the proportion of components have a large impact on the modification results.

Meanwhile, the effect of the star SBS modifier on the penetration is significantly smaller than that of the linear modifier, while the improvement of softening point of star SBS is larger than that of linear SBS. it is clear from the comparison that the dissolution of SBS modifier occurs after absorbing the light components in asphalt, and the modification effect of star modifier is better. Although the star modifier has a complex molecular structure, large molecular weight and strong intermolecular forces, resulting in poor solubility and a more difficult reaction with the lighter components in the asphalt compared to the linear SBS modifier, the modified asphalt will perform better strength and stability than the linear modified asphalt once the star modifier reacts completely with the components in the asphalt to form a stable system. 

## 3. Artificial Neural Networks (ANNs)

### 3.1. Background

ANN is a kind of information processing method based on human brain biological system. The importance is constructing a new structure of an information processing system. ANN operates together through a great deal of highly interconnected processing neurons to work out issues [1].

Neurons are connected by connecting links. Each link has a weight multiplied by the transmission signal, and the output of each neuron can be confirmed by the activation function. At present, there are many methods to activate functions—sigmoid is usually used to activate nonlinear functions—and the mapping relationship between functions is established by a neural network. New results can be produced by importing unknown inputs into the network model [24].

There are many kinds of efficient algorithms for the model training of neural networks. Most of these algorithms can be seen as a straight-forward application of statistical estimation and optimization theory. Some form of gradient descent is usually used by most of the algorithms. It is completed by taking the derivative of the cost function about the network parameters and then changing those indexes in a gradient relevant direction. Among them, the back propagation algorithm is the most widely used. 

**Figure 4:**
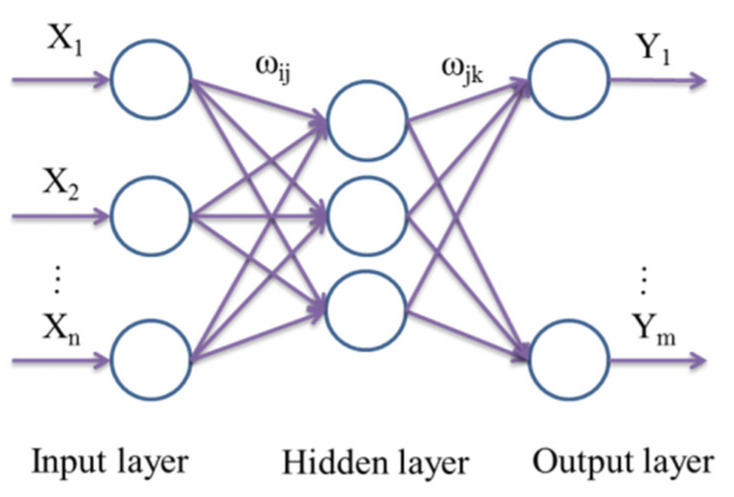
Topological structure of neural network.

The BP neural network is a kind of multilayer feed-forward neural network. The specific topological structure of the BP neural network is shown in Figure 4. Neurons are arranged in each layer. The layers between the input signal applied to the input layer and the output layer that contributes to the output signal are called the hidden layer.

If the neural network model is considered as a nonlinear function, the input values X_1_, X_2_,… X_n_ and output values Y_1_,…, Y_n_ are the independent variables and dependent variables, respectively. ω_ij_ and ω_jk_ are the weight coefficients of the neural network. When the number of input and output nodes are n and m, the function mapping relationship of BP neural network changes from n independent variables to m dependent variables. 

Before prediction by the neural network model, the network should be trained to have an associative memory and prediction capacity. The training of the BP neural network mainly consists of the following procedures:

(1) Network initialization. In accordance with the input and output of the system (X,Y), we determine the number of input layer nodes n, the number of hidden layer nodes l, the number of output layer nodes m, and the connection weight *ω_ij_* and *ω_jk_*, initializing the threshold a of hidden layer and the threshold b of output layer.

(2) Hidden layer output calculation. In accordance with *X*, *ω_ij_*, *a*, we calculate the hidden layer output H.

(1) $$H_{j}=f\left(\sum_{i=1}^{n}\omega_{ij}x_{i}-a_{j}\right)\;j=1,2,\cdots ,l$$

where f is the activation function of the hidden layer, the function has multiple expressions and can be chosen according to the specific situation.

(3) Output layer output calculation. In accordance with *H*, *ω_jk_*, *b*, we calculate the output layer output *O*.

(2) $$O_{k}=\sum_{j=1}^{l}H_{j}\omega_{jk}-b_{k}\;k=1,2,\cdots ,m$$

(4) Error calculation. In accordance with the prediction output *O* and the desired output *Y*, we calculate the error e.

(3) $$e_{k}=Y_{k}-O_{k}$$

(5) Weight update. In accordance with the error *e*, we renew the network connection weights *ω_ij_* and *ω_jk_*.

(4) $$\omega_{\mathrm{ij}}=\omega_{\mathrm{ij}}+\eta H_{j}\left(1-H_{j}\right)x\left(i\right)\sum_{k=1}^{m}\omega_{jk}e_{k}\;i=1,2,\cdots ,n$$

(5) $$\omega_{\mathrm{jk}}=\omega_{jk}+\eta H_{j}e_{k}$$

where, η is learning rate.

(6) Threshold update. In accordance with the error e, we renew the network node threshold *a* and *b*.

(6) $$a_{j}=a_{j}+\eta H_{j}\left(1-H_{j}\right)\sum_{k=1}^{m}\omega_{jk}e_{k}$$

(7) $$b_{k}=b_{k}+e_{k}$$

(7) Identify if the algorithm iteration converges, and if not, back to step 2.

### 3.2. Model Construction 

Typically, a multilayer feed-forward neural network is constructed by an input layer, output layer, and one or more hidden layers. In the work, the input layer is ATR-FTIR data of all prepared samples, while the output layer is the performance parameters such as SBS content, penetration and softening point.

ANNs model is proposed to determine the performance parameters of SBS modified asphalt produced by 30 samples. For the purpose of evaluating the prediction effect of the model, some statistical approaches such as the coefficient of determination (R^2^), the root mean square error (R_MSE_), and the coefficient of variation (COV) can be adopted to compare the difference between predicted and real values.

(8) $$R^{2}=1-\frac{\sum_{i=1}^{n}{\left(y_{i}^{p}-y_{i}^{m}\right)}^{2}}{\sum_{i=1}^{n}{\left(y_{i}^{m}\right)}^{2}}$$

(9) $$R_{MSE}=\sqrt{\frac{\sum_{i=1}^{n}{\left(y_{i}^{p}-y_{i}^{m}\right)}^{2}}{n}}$$

(10) $$\mathrm{COV}=\frac{R_{MSE}}{\left|t_{i}^{m}\right|}\times 100$$

where, n is the number of date patterns in the independent data set, $y_{i}^{p}$ is the predicted value, $y_{i}^{m}$ is the measured value, $t_{i}^{m}$ is the mean value of all measured data.

The back propagation learning algorithm has been applied in a feed-forward, single hidden layer neural network. Three variants of the BP algorithm are used in this research, including the Levenberg -Marquardt (L-M), scaled conjugate gradient (SCG), and Bayesian Regularization (BR) algorithms, respectively. Each variation of BP training algorithms has certain advantages and disadvantages depending on the network architecture and the complexity of the specific problem. Currently, it is difficult to identify which algorithm is optimal for a particular issue, therefore, the algorithm is normally chosen by trial and error.

During the training process, the quantities of neurons 3, 5, and 7 were applied in the hidden layer to identify the output exactly. The data set consisted of 150 data patterns obtained by tests. A total of 104 data patterns (70% of the total number) were randomly chosen for training the network model, 23 patterns (30%) were used for validation, and the other 23 patterns (30%) were used as the test data sets. Validation data is used to assist model building. Test data is used to detect the model building and evaluate the accuracy of the model.

### 3.3. Data Preprocessing 

**Figure 5:**
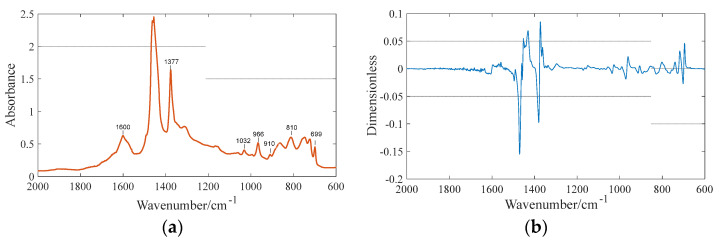
Infrared spectrum of SBS modified asphalt. The spectrum before (**a**)and after (**b**) processing.

As shown in Table 3, there are many existing infrared data preprocessing methods to eliminate the random errors that occur during the data acquisition. Based on the comparative analysis of various preprocessing methods, the first-order derivative and smoothing algorithms are selected for the subsequent preprocessing of infrared data in this paper. The former has the advantages of eliminating baseline interference, improving spectral resolution and facilitating the analysis of overlapping peaks. The latter can eliminate the effect of noise on the spectra [7]. The spectral images of SBS modified asphalt before and after preprocessing are presented in Figure 5. 

### 3.4. Model Optimization

Based on the existence of unsatisfactory prediction accuracy of the pre-processed ANN model, this paper achieves the determination of specific components within the characteristic spectral interval by shortening and eliminating the training time of the spectral interval and constructing a prediction model adapted to the characteristic spectral interval. Moreover, the selection of wavelength range should consider chemical learning, for example, the relationship between the performance indicators and characteristic functional groups, and the degree of correlation between characteristics parameters and pretreatment spectra.

**Figure 6:**
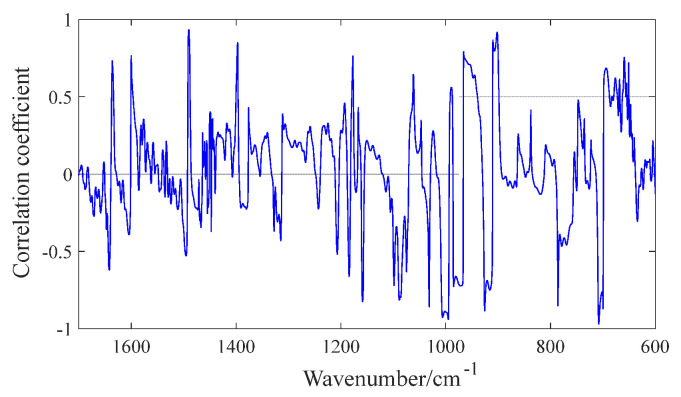
Correlation between IR and SBS content.

The dependency relation between the SBS content and the preprocessing spectrum of all samples is shown in Figure 6. It can be figured out that SBS content and spectrum value have better correlation coefficients in the wavelength range from 690~1100 cm^−1^. Among this area, the structural characteristic peaks of trans butadiene are 966 cm^−1^ and 910 cm^−1^. The single replaced peak of styryl benzene ring is 699 cm^−1^. Therefore, the peaks of trans butadiene and styryl benzene ring are also that of SBS modified materials. Subsequently, the sulfoxide base S = O has a stretching vibration absorption peak of 1032 cm^−1^. Moreover, 720 cm^−1^ is the flexural vibration peak of alkanes representing asphalt saturated hydrocarbon components. 740~840 cm^−1^ is the benzene vibration peak representing the aromatic component of asphalt. Based on the integrated thoughts of chemical knowledge mechanism and correlation coefficient, 680~720 cm^−1^ and 880~1050 cm^−1^ were identified as the characteristic intervals of SBS content prediction model.

In order to verify the correctness of the model optimization method, the prediction models were constructed in different wavelength ranges and sensitivity analysis was carried out as well; the results are displayed in Table 4. Through the results, it can be seen that the prediction accuracy in the range of 4000–400 cm^−1^ cannot meet the requirements, and when the wavelength range is shortened to 1300–650 cm^−1^, 720–680 cm^−1^ and 1050–880 cm^−1^, the precision of the prediction reaches a high level, which indicates that the model optimization by shortening the wavelength range is feasible.

Although the softening point and penetration of asphalt are physical indicators, they are closely related to the chemical components. Thus, it is feasible to use infrared spectroscopy as a quantitative analysis tool for the information carrier to determine the properties of asphalt.

Based on the literature [22], it is known that the characteristic peaks of the chemical components in the modifier are the basis for determining the wavelength range of the prediction model. Ultimately, 3100~2800 cm^−1^, 1650~1300 cm^−1^ and 1050~680 cm^−1^ were used as the wavelength interval ranges for the penetration prediction model, while 1700~1300 cm^−1^ and 1050~680 cm^−1^ were used for the softening point prediction model.

## 4. Results and Discussion

### 4.1. SBS Content Prediction Model

Based on the previous study, we have determined that as the number of neurons increases, the iterations do not show a trend of first increasing and then decreasing, and the prediction error decreases. In this paper, the Levenberg-Marquardt algorithm, Bayesian Regularization algorithm and Scaled Conjugate Gradient algorithm and the number of hidden neurons in layers 3, 5 and 7 were used to obtain the best model. The results are shown in Table 5.

**Figure 7:**
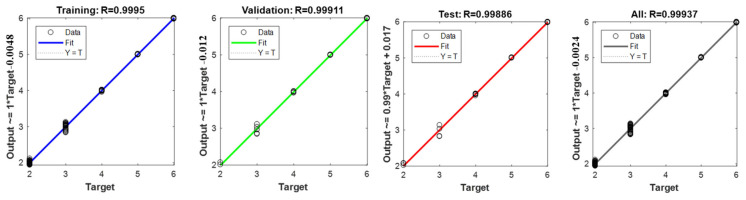
SBS content Regression model results of L-M algorithm with seven hidden neurons.

The results in Table 4 show that the R^2^ in the calculation results with different algorithms and number of neurons ranged from 0.9987 to 09958, and the root mean square coefficient ranged from 0.0025 to 0.0086, and the differences were not significant. It indicates that the different training algorithms and the number of hidden neurons have little effect on the prediction accuracy of the model, and the relationship between the number of neurons and the prediction accuracy is not simply linear, or perhaps the algorithms selected in this paper are well-suited and do not have a significant effect on the model results. However, the L-M algorithm with seven hidden neurons obtained the best model for SBS content prediction in the tested range, as in Figure 7.

The prediction accuracy of this method is consistent with the traditional standard curve method which can satisfy a high demand. Concurrently, the applicability of the new method is also wider because of the diversity of asphalt types in the modeling process.

The correlation coefficients between the prediction models and the actual values can reach 0.941 and 0.993, respectively, in the existing literature on the determination of modifier content in modified asphalt using artificial neural networks and ATR-FTIR, which meet the requirements of experimental accuracy [25,26]. However, the above two methods are only for one kind of asphalt and modifier, and the model applicability is poor and cannot be widely used in practice.

### 4.2. The Softening Point and Penetration Prediction Model

By comparing the accuracy of the prediction models constructed under the L-M algorithm with seven hidden neurons based on the number of different asphalt types, it can be seen that the prediction accuracy of the model gradually decreases as the asphalt type increases. Table 6 shows the prediction model data of the penetration and softening point of modified asphalt prepared from different asphalts, and the analysis results show that the influence of asphalt type on the prediction model of softening point is lower than that on the penetration.

By comparing the accuracy of the prediction models of penetration, softening point and SBS modifier doping, it can be seen that the prediction accuracy of SBS modifier doping is better than the first two. It may be that the experimental input data of penetration and softening point have errors, which accumulate and have a greater influence on the prediction results. Therefore, the wavelength range of the prediction model can be subsequently determined by SBS characteristic peaks, which can reduce the noise of the model system and improve the accuracy of the model to meet the applicability and accuracy of the model as well.

## 5. Conclusions

In this paper, a prediction model of SBS modified asphalt was constructed based on ATR-FTIR database, and the parameters of SBS admixture, penetration and softening point of modified asphalt were determined rapidly by an artificial neural network. The R^2^, R_MSE_ and coefficient of variation were used to evaluate the differences between the predicted and actual values. According to the prediction results, it can be seen that the model predicts the SBS modifier satisfactorily, with R^2^ reaching 0.9987 and R_MSE_ 0.0025 under the L-M algorithm with seven hidden neurons; the accuracy of penetration and softening point of single modified asphalt can also meet the requirements, with R^2^ of 0.982 and 0.964, which can meet the demand for rapid determination of modified asphalt properties in practical engineering.

However, there are large differences in the properties of different grades of asphalt, and the established prediction model cannot achieve the prediction of the properties of different PG grades of modified asphalt. It is necessary to extend the existing infrared spectral data and improve the applicability of the existing model through new machine learning algorithms, in the hope of improving the accuracy of the performance prediction of different types of asphalt.

## Figures and Tables

**Table 1:** Basic technical indicators of matrix asphalt.

Category	Penetration (25 °C, 0.1 mm)	Ductility (15 °C)/cm	Softening Poi1nt/°C	Viscosity (135 °C)/mPa s	Viscosity (175 °C)/mPa·s
Sinopec	56.0	>150	50.5	473.4	90.2
Cnooc	67.0	>150	49.3	435.1	84.7
Shell	75.3	>100	49.9	422.1	83.0

**Table 2:** SBS Performance Index.

Performance Indicators	Unit	Test Results
Ash	%	0.16
300% tensile stress	MPa	2.4
Volatile fraction	%	0.88
Melt flow rate	g/(10 min)	0.03
Tear elongation	%	720

**Table 3:** Contrast of varying methods.

Methods	R_MSE_	R^2^	COV
1st derivative	0.031	0.921	0.80
2nd derivative	0.040	0.862	1.02
Smoothing	0.026	0.944	0.67
1st derivative + Smoothing	0.010	0.993	0.29

**Table 4:** Sensitivity analysis for the impact of wavelength range.

Modeling Interval	R_MSE_	R^2^	COV
400~4000 cm^−1^	0.15	0.73	3.8
650~1300 cm^−1^	0.02	0.97	0.5
680~720 cm^−1^ and 880~1050 cm^−1^	0.01	0.99	0.25

**Table 5:** Parameter results for SBS content model.

Training Algorithm	Number of Hidden Neurons	R^2^	R_MSE_
L-M	7	0.9987	0.0025
L-M	5	0.9986	0.0027
L-M	3	0.9982	0.0035
B-R	7	0.9982	0.0035
B-R	5	0.9982	0.003
B-R	3	0.9982	0.0036
SCG	7	0.9946	0.0106
SCG	5	0.9976	0.0048
SCG	3	0.9958	0.0086

**Table 6:** Parameters results for softening point and penetration model.

Model Type	Modeling Interval (cm^−1^)	Number of Asphalt Type	Sample Size	R^2^	R_MSE_
penetration prediction model	3100~2800, 1650~1300, 1050~680	1	50	0.982	1.56
		2	100	0.977	2.45
		3	150	0.922	6.63
Softening point prediction model	1700~1300, 1050~680	1	50	0.964	3.36
		2	100	0.96	3.07
		3	150	0.945	4.35

## Data Availability

Some or all data, models, or code that support the findings of this study are available from the corresponding author upon reasonable request.
